# 5-Bromo-2-(4-fluoro­phen­yl)-7-methyl-3-methyl­sulfinyl-1-benzofuran

**DOI:** 10.1107/S160053680905418X

**Published:** 2009-12-19

**Authors:** Hong Dae Choi, Pil Ja Seo, Byeng Wha Son, Uk Lee

**Affiliations:** a Department of Chemistry, Dongeui University, San 24 Kaya-dong Busanjin-gu, Busan 614-714, Republic of Korea; b Department of Chemistry, Pukyong National University, 599-1 Daeyeon 3-dong, Nam-gu, Busan 608-737, Republic of Korea

## Abstract

In the title compound, C_16_H_12_BrFO_2_S, the O atom and the methyl group of the methyl­sulfinyl substituent are located on opposite sides of the plane through the benzofuran fragment. The 4-fluoro­phenyl ring is rotated out of the benzofuran plane, as indicated by the dihedral angle of 16.17 (5)°. The crystal structure exhibits an inter­molecular C—H⋯O hydrogen bond and a Br⋯O halogen inter­action [3.112 (2) Å].

## Related literature

For the crystal structures of similar 2-(4-fluoro­phen­yl)-5-halo-3-methyl­sulfinyl-1-benzofuran derivatives, see: Choi *et al.* (2009*a* ▶,*b* ▶, 2010 ▶). For the pharmacological activity of benzofuran compounds, see: Howlett *et al.* (1999 ▶); Twyman & Allsop (1999 ▶). For natural products with benzofuran rings, see: Akgul & Anil (2003 ▶); Soekamto *et al.* (2003 ▶). For a review of halogen inter­actions, see: Politzer *et al.* (2007 ▶).
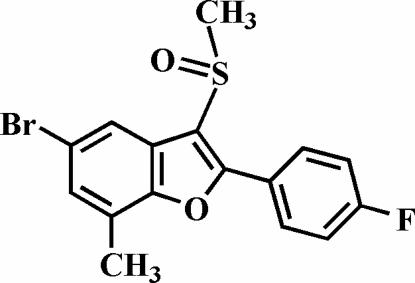


## Experimental

### 

#### Crystal data

C_16_H_12_BrFO_2_S
*M* *_r_* = 367.23
Triclinic, 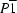

*a* = 7.5313 (6) Å
*b* = 9.8089 (7) Å
*c* = 10.9117 (8) Å
α = 106.567 (1)°
β = 92.634 (1)°
γ = 109.526 (1)°
*V* = 719.23 (9) Å^3^
*Z* = 2
Mo *K*α radiation
μ = 3.01 mm^−1^
*T* = 173 K
0.60 × 0.40 × 0.20 mm

#### Data collection

Bruker SMART APEXII CCD diffractometer
Absorption correction: multi-scan (*SADABS*; Bruker, 2009 ▶) *T*_min_ = 0.586, *T*_max_ = 0.746
6248 measured reflections
3083 independent reflections
2802 reflections with *I* > 2σ(*I*)
*R*_int_ = 0.016

#### Refinement

*R*[*F* ^2^ > 2σ(*F* ^2^)] = 0.023
*wR*(*F* ^2^) = 0.060
*S* = 1.05
3083 reflections
192 parameters
H-atom parameters constrained
Δρ_max_ = 0.34 e Å^−3^
Δρ_min_ = −0.39 e Å^−3^

### 

Data collection: *APEX2* (Bruker, 2009 ▶); cell refinement: *SAINT* (Bruker, 2009 ▶); data reduction: *SAINT*; program(s) used to solve structure: *SHELXS97* (Sheldrick, 2008 ▶); program(s) used to refine structure: *SHELXL97* (Sheldrick, 2008 ▶); molecular graphics: *ORTEP-3* (Farrugia, 1997 ▶) and *DIAMOND* (Brandenburg, 1998 ▶); software used to prepare material for publication: *SHELXL97*.

## Supplementary Material

Crystal structure: contains datablocks global, I. DOI: 10.1107/S160053680905418X/kp2245sup1.cif

Structure factors: contains datablocks I. DOI: 10.1107/S160053680905418X/kp2245Isup2.hkl

Additional supplementary materials:  crystallographic information; 3D view; checkCIF report

## Figures and Tables

**Table 1:** Hydrogen-bond geometry (Å, °)

*D*—H⋯*A*	*D*—H	H⋯*A*	*D*⋯*A*	*D*—H⋯*A*
C15—H15*C*⋯O2^i^	0.96	2.58	3.294 (2)	131

Symmetry code: (i) 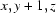.

**Fig. 1.**
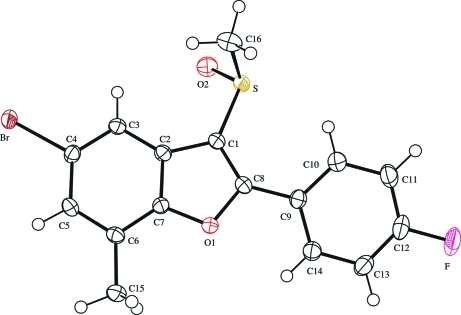
The molecular structure of the title compound with the atom numbering scheme. Displacement ellipsoids are drawn at the 50% probability level. H atoms are presented as a small spheres of arbitrary radius.

**Fig. 2.**
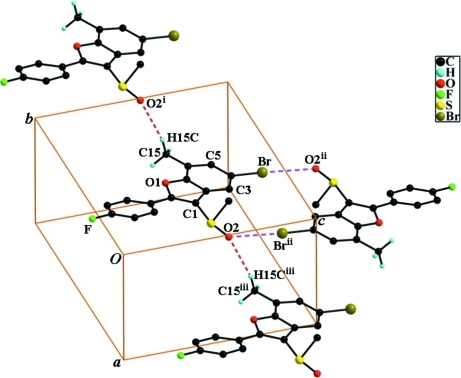
C—H···O and C—Br···O interactions (dotted lines) in the crystal structure of the title compound. [Symmetry codes: (i) x, y + 1, z; (ii) - x + 1, - y + 1, - z + 2; (iii) x, y - 1, z .]

## supplementary crystallographic information

### Comment

Molecules of benzofuran ring skeleton have attracted considerable interest, on account of their pharmacological activity (Howlett *et al.*, 1999; Twyman & Allsop, 1999) and their occurrence as natural products (Akgul & Anil, 2003; Soekamto *et al.*, 2003). As a part of our continuing studies on the effect of side chain substituents on the solid state structures of 2-(4-fluorophenyl)-5-halo-3-methylsulfinyl-1-benzofuran analogues (Choi *et al.*, 2009*a*, *b*, 2010), we report the crystal structure of the title compound (Fig. 1).

The benzofuran unit is essentially planar, with a mean deviation of 0.014 (1) Å from the least-squares plane defined by the nine constituent atoms. The dihedral angle formed by the plane of the benzofuran and the 4-fluorophenyl ring is 16.17 (5)°. The crystal packing (Fig. 2) is stabilized by an intermolecular C—H···O hydrogen bond between the methyl H atom and the oxygen of the S═O unit, with a C15—H15C···O2^i^ (Table 1and Fig. 2). The contact C-Br···O  involving the the oxygen atom of the S═O unit [Br···O2^ii^ = 3.112 (1) Å; C—Br···O2^ii^ = 173.44 (7)°] is significantly shorter than the sum of van der Waals radia (3.40 Å) (Politzer *et al.*, 2007).

### Experimental

77% 3-Chloroperoxybenzoic acid (247 mg, 1.1 mmol) was added in small portions to a stirred solution of 5-bromo-2-(4-fluorophenyl)-7-methyl-3-methylsulfanyl-1-benzofuran (351 mg, 1.0 mmol) in dichloromethane (30 mL) at 273 K. After being stirred at room temperature for 3 h, the mixture was washed with saturated sodium bicarbonate solution and the organic layer was separated, dried over magnesium sulfate, filtered and concentrated in vacuum. The residue was purified by column chromatography (hexane–ethyl acetate, 1:1 v/v) to afford the title compound as a colourless solid [yield 85%, m.p. 477–478 K; *R*_f_ = 0.71 (hexane–ethyl acetate, 1:1 v/v)]. Single crystals suitable for X-ray diffraction were prepared by slow evaporation of a solution of the title compound in tetrahydrofuran at room temperature.

### Refinement

All H atoms were positioned geometrically and refined using a riding model, with C—H = 0.93 Å for aromatic H atoms and 0.96 Å for methyl H atoms, and with *U*_iso_(H) = 1.2*U*_eq_(C) for aromatic H atoms and 1.5*U*_eq_(C) for methyl H atoms.

### Crystal data

**Table:**

C_16_H_12_BrFO_2_S	*Z* = 2
*M**_r_* = 367.23	*F*(000) = 368
Triclinic, *P*1	*D*_x_ = 1.696 Mg m^−^^3^
Hall symbol: -P 1	Mo *K*α radiation, λ = 0.71073 Å
*a* = 7.5313 (6) Å	Cell parameters from 4231 reflections
*b* = 9.8089 (7) Å	θ = 2.3–27.4°
*c* = 10.9117 (8) Å	µ = 3.01 mm^−^^1^
α = 106.567 (1)°	*T* = 173 K
β = 92.634 (1)°	Block, colourless
γ = 109.526 (1)°	0.60 × 0.40 × 0.20 mm
*V* = 719.23 (9) Å^3^	

### Data collection

**Table:**

Bruker SMART APEXII CCD diffractometer	3083 independent reflections
Radiation source: Rotating Anode	2802 reflections with *I* > 2σ(*I*)
HELIOS	*R*_int_ = 0.016
Detector resolution: 10.0 pixels mm^-1^	θ_max_ = 27.0°, θ_min_ = 2.0°
φ and ω scans	*h* = −9→9
Absorption correction: multi-scan (*SADABS*; Bruker, 2009)	*k* = −12→12
*T*_min_ = 0.586, *T*_max_ = 0.746	*l* = −13→13
6248 measured reflections	

### Refinement

**Table:**

Refinement on *F*^2^	Primary atom site location: structure-invariant direct methods
Least-squares matrix: full	Secondary atom site location: difference Fourier map
*R*[*F*^2^ > 2σ(*F*^2^)] = 0.023	Hydrogen site location: difference Fourier map
*wR*(*F*^2^) = 0.060	H-atom parameters constrained
*S* = 1.05	*w* = 1/[σ^2^(*F*_o_^2^) + (0.0302*P*)^2^ + 0.3573*P*] where *P* = (*F*_o_^2^ + 2*F*_c_^2^)/3
3083 reflections	(Δ/σ)_max_ < 0.001
192 parameters	Δρ_max_ = 0.34 e Å^−^^3^
0 restraints	Δρ_min_ = −0.39 e Å^−^^3^

### Special details

**Table:**

Geometry. All esds (except the esd in the dihedral angle between two l.s. planes) are estimated using the full covariance matrix. The cell esds are taken into account individually in the estimation of esds in distances, angles and torsion angles; correlations between esds in cell parameters are only used when they are defined by crystal symmetry. An approximate (isotropic) treatment of cell esds is used for estimating esds involving l.s. planes.
Refinement. Refinement of F^2^ against ALL reflections. The weighted R-factor wR and goodness of fit S are based on F^2^, conventional R-factors R are based on F, with F set to zero for negative F^2^. The threshold expression of F^2^ > 2sigma(F^2^) is used only for calculating R-factors(gt) etc. and is not relevant to the choice of reflections for refinement. R-factors based on F^2^ are statistically about twice as large as those based on F, and R- factors based on ALL data will be even larger.

### Fractional atomic coordinates and isotropic or equivalent isotropic displacement parameters (Å^2^)

**Table:**

	*x*	*y*	*z*	*U*_iso_*/*U*_eq_	
Br	0.56892 (3)	0.75819 (2)	1.066631 (17)	0.02668 (7)	
S	0.18420 (7)	0.22498 (5)	0.55788 (4)	0.02381 (11)	
O1	0.31077 (18)	0.62985 (14)	0.50645 (12)	0.0214 (3)	
O2	0.3359 (2)	0.21723 (16)	0.64548 (14)	0.0320 (3)	
F	−0.0757 (2)	0.21942 (17)	−0.05951 (11)	0.0476 (4)	
C1	0.2455 (3)	0.4180 (2)	0.56565 (17)	0.0207 (4)	
C2	0.3406 (3)	0.5483 (2)	0.67945 (17)	0.0197 (3)	
C3	0.3970 (3)	0.5713 (2)	0.81000 (17)	0.0217 (4)	
H3	0.3726	0.4901	0.8427	0.026*	
C4	0.4906 (3)	0.7202 (2)	0.88760 (17)	0.0216 (4)	
C5	0.5318 (3)	0.8455 (2)	0.84171 (18)	0.0217 (4)	
H5	0.5973	0.9434	0.8985	0.026*	
C6	0.4760 (3)	0.8255 (2)	0.71248 (18)	0.0207 (4)	
C7	0.3798 (2)	0.6748 (2)	0.63653 (17)	0.0193 (3)	
C8	0.2316 (2)	0.4724 (2)	0.46474 (18)	0.0203 (4)	
C9	0.1517 (3)	0.4038 (2)	0.32705 (17)	0.0215 (4)	
C10	0.0209 (3)	0.2541 (2)	0.27673 (19)	0.0283 (4)	
H10	−0.0167	0.1961	0.3316	0.034*	
C11	−0.0534 (3)	0.1909 (3)	0.1460 (2)	0.0332 (5)	
H11	−0.1376	0.0903	0.1121	0.040*	
C12	0.0004 (3)	0.2802 (3)	0.06824 (19)	0.0318 (5)	
C13	0.1276 (3)	0.4285 (3)	0.11316 (19)	0.0298 (4)	
H13	0.1607	0.4860	0.0575	0.036*	
C14	0.2051 (3)	0.4901 (2)	0.24326 (19)	0.0252 (4)	
H14	0.2933	0.5895	0.2752	0.030*	
C15	0.5111 (3)	0.9548 (2)	0.65832 (19)	0.0268 (4)	
H15A	0.5531	0.9286	0.5760	0.040*	
H15B	0.6076	1.0448	0.7168	0.040*	
H15C	0.3952	0.9737	0.6475	0.040*	
C16	−0.0164 (3)	0.2099 (3)	0.6426 (2)	0.0351 (5)	
H16A	−0.0639	0.1111	0.6538	0.053*	
H16B	−0.1150	0.2230	0.5936	0.053*	
H16C	0.0222	0.2874	0.7258	0.053*	

### Atomic displacement parameters (Å^2^)

**Table:**

	*U*^11^	*U*^22^	*U*^33^	*U*^12^	*U*^13^	*U*^23^
Br	0.03804 (12)	0.02214 (11)	0.01767 (10)	0.01046 (8)	−0.00056 (7)	0.00439 (7)
S	0.0331 (3)	0.0152 (2)	0.0220 (2)	0.00897 (19)	0.00200 (19)	0.00445 (17)
O1	0.0269 (7)	0.0172 (6)	0.0193 (6)	0.0071 (5)	0.0009 (5)	0.0062 (5)
O2	0.0397 (8)	0.0274 (8)	0.0342 (8)	0.0167 (7)	0.0012 (6)	0.0128 (6)
F	0.0504 (8)	0.0559 (9)	0.0194 (6)	0.0055 (7)	−0.0086 (6)	0.0053 (6)
C1	0.0239 (9)	0.0164 (8)	0.0205 (9)	0.0066 (7)	0.0025 (7)	0.0052 (7)
C2	0.0223 (8)	0.0170 (8)	0.0208 (9)	0.0087 (7)	0.0039 (7)	0.0055 (7)
C3	0.0282 (9)	0.0175 (9)	0.0211 (9)	0.0095 (7)	0.0039 (7)	0.0075 (7)
C4	0.0260 (9)	0.0226 (9)	0.0174 (8)	0.0112 (7)	0.0019 (7)	0.0058 (7)
C5	0.0232 (9)	0.0159 (9)	0.0232 (9)	0.0071 (7)	0.0020 (7)	0.0026 (7)
C6	0.0222 (9)	0.0170 (9)	0.0239 (9)	0.0083 (7)	0.0051 (7)	0.0065 (7)
C7	0.0211 (8)	0.0186 (9)	0.0186 (8)	0.0076 (7)	0.0015 (7)	0.0062 (7)
C8	0.0200 (8)	0.0173 (9)	0.0221 (9)	0.0063 (7)	0.0034 (7)	0.0046 (7)
C9	0.0203 (8)	0.0242 (9)	0.0202 (9)	0.0095 (7)	0.0020 (7)	0.0059 (7)
C10	0.0276 (10)	0.0276 (10)	0.0253 (10)	0.0051 (8)	0.0010 (8)	0.0083 (8)
C11	0.0282 (10)	0.0311 (11)	0.0287 (11)	0.0026 (9)	−0.0032 (8)	0.0033 (9)
C12	0.0290 (10)	0.0415 (12)	0.0187 (9)	0.0108 (9)	−0.0018 (8)	0.0037 (9)
C13	0.0310 (10)	0.0368 (12)	0.0233 (10)	0.0117 (9)	0.0031 (8)	0.0130 (9)
C14	0.0259 (9)	0.0247 (10)	0.0242 (9)	0.0093 (8)	0.0022 (8)	0.0070 (8)
C15	0.0364 (11)	0.0192 (9)	0.0261 (10)	0.0102 (8)	0.0063 (8)	0.0087 (8)
C16	0.0333 (11)	0.0280 (11)	0.0443 (13)	0.0068 (9)	0.0101 (10)	0.0164 (10)

### Geometric parameters (Å, °)

**Table:**

Br—C4	1.907 (2)	C6—C15	1.497 (3)
Br—O2^i^	3.112 (2)	C8—C9	1.463 (2)
S—O2	1.491 (2)	C9—C10	1.397 (3)
S—C1	1.768 (2)	C9—C14	1.401 (3)
S—C16	1.794 (2)	C10—C11	1.386 (3)
O1—C7	1.379 (2)	C10—H10	0.9300
O1—C8	1.381 (2)	C11—C12	1.368 (3)
F—C12	1.360 (2)	C11—H11	0.9300
C1—C8	1.368 (3)	C12—C13	1.375 (3)
C1—C2	1.445 (2)	C13—C14	1.386 (3)
C2—C7	1.394 (2)	C13—H13	0.9300
C2—C3	1.399 (2)	C14—H14	0.9300
C3—C4	1.378 (3)	C15—H15A	0.9600
C3—H3	0.9300	C15—H15B	0.9600
C4—C5	1.403 (3)	C15—H15C	0.9600
C5—C6	1.391 (3)	C16—H16A	0.9600
C5—H5	0.9300	C16—H16B	0.9600
C6—C7	1.389 (2)	C16—H16C	0.9600
			
C4—Br—O2^i^	173.44 (7)	C10—C9—C8	121.62 (17)
O2—S—C1	107.33 (9)	C14—C9—C8	119.58 (17)
O2—S—C16	105.95 (10)	C11—C10—C9	120.85 (19)
C1—S—C16	97.80 (9)	C11—C10—H10	119.6
C7—O1—C8	106.74 (13)	C9—C10—H10	119.6
C8—C1—C2	107.32 (16)	C12—C11—C10	118.3 (2)
C8—C1—S	127.08 (14)	C12—C11—H11	120.8
C2—C1—S	125.27 (14)	C10—C11—H11	120.8
C7—C2—C3	118.96 (16)	F—C12—C11	118.39 (19)
C7—C2—C1	105.04 (15)	F—C12—C13	118.55 (19)
C3—C2—C1	136.00 (17)	C11—C12—C13	123.06 (19)
C4—C3—C2	116.62 (16)	C12—C13—C14	118.48 (19)
C4—C3—H3	121.7	C12—C13—H13	120.8
C2—C3—H3	121.7	C14—C13—H13	120.8
C3—C4—C5	123.42 (17)	C13—C14—C9	120.46 (18)
C3—C4—Br	118.40 (14)	C13—C14—H14	119.8
C5—C4—Br	118.17 (14)	C9—C14—H14	119.8
C6—C5—C4	120.97 (17)	C6—C15—H15A	109.5
C6—C5—H5	119.5	C6—C15—H15B	109.5
C4—C5—H5	119.5	H15A—C15—H15B	109.5
C7—C6—C5	114.57 (16)	C6—C15—H15C	109.5
C7—C6—C15	121.86 (17)	H15A—C15—H15C	109.5
C5—C6—C15	123.54 (17)	H15B—C15—H15C	109.5
O1—C7—C6	124.01 (16)	S—C16—H16A	109.5
O1—C7—C2	110.54 (15)	S—C16—H16B	109.5
C6—C7—C2	125.44 (17)	H16A—C16—H16B	109.5
C1—C8—O1	110.33 (16)	S—C16—H16C	109.5
C1—C8—C9	135.27 (17)	H16A—C16—H16C	109.5
O1—C8—C9	114.40 (15)	H16B—C16—H16C	109.5
C10—C9—C14	118.79 (17)		
			
O2—S—C1—C8	138.31 (17)	C1—C2—C7—O1	1.57 (19)
C16—S—C1—C8	−112.23 (18)	C3—C2—C7—C6	1.6 (3)
O2—S—C1—C2	−34.18 (18)	C1—C2—C7—C6	−177.92 (17)
C16—S—C1—C2	75.29 (18)	C2—C1—C8—O1	−0.4 (2)
C8—C1—C2—C7	−0.7 (2)	S—C1—C8—O1	−173.93 (13)
S—C1—C2—C7	172.99 (14)	C2—C1—C8—C9	−179.25 (19)
C8—C1—C2—C3	179.9 (2)	S—C1—C8—C9	7.2 (3)
S—C1—C2—C3	−6.4 (3)	C7—O1—C8—C1	1.32 (19)
C7—C2—C3—C4	−0.6 (3)	C7—O1—C8—C9	−179.53 (14)
C1—C2—C3—C4	178.7 (2)	C1—C8—C9—C10	17.4 (3)
C2—C3—C4—C5	−0.7 (3)	O1—C8—C9—C10	−161.47 (17)
C2—C3—C4—Br	179.49 (13)	C1—C8—C9—C14	−163.3 (2)
C3—C4—C5—C6	1.1 (3)	O1—C8—C9—C14	17.8 (2)
Br—C4—C5—C6	−179.08 (14)	C14—C9—C10—C11	0.7 (3)
C4—C5—C6—C7	−0.2 (3)	C8—C9—C10—C11	180.00 (18)
C4—C5—C6—C15	178.35 (17)	C9—C10—C11—C12	−1.8 (3)
C8—O1—C7—C6	177.69 (17)	C10—C11—C12—F	−178.57 (19)
C8—O1—C7—C2	−1.81 (19)	C10—C11—C12—C13	1.4 (3)
C5—C6—C7—O1	179.43 (16)	F—C12—C13—C14	−179.89 (18)
C15—C6—C7—O1	0.9 (3)	C11—C12—C13—C14	0.1 (3)
C5—C6—C7—C2	−1.1 (3)	C12—C13—C14—C9	−1.3 (3)
C15—C6—C7—C2	−179.68 (17)	C10—C9—C14—C13	0.8 (3)
C3—C2—C7—O1	−178.94 (15)	C8—C9—C14—C13	−178.44 (17)

Symmetry codes: (i) −*x*+1, −*y*+1, −*z*+2.

### Hydrogen-bond geometry (Å, °)

**Table:**

*D*—H···*A*	*D*—H	H···*A*	*D*···*A*	*D*—H···*A*
C15—H15C···O2^ii^	0.96	2.58	3.294 (2)	131

Symmetry codes: (ii) *x*, *y*+1, *z*.
